# Drug-Resistant Tuberculosis Among Children: A Systematic Review and Meta-Analysis

**DOI:** 10.3389/fpubh.2021.721817

**Published:** 2021-08-18

**Authors:** Wan-mei Song, Yi-fan Li, Yun-xia Liu, Yao Liu, Chun-bao Yu, Jin-yue Liu, Huai-chen Li

**Affiliations:** ^1^Department of Respiratory and Critical Care Medicine, Shandong Provincial Hospital Affiliated to Shandong University, Shandong Provincial Hospital Affiliated to Shandong First Medical University, Jinan, China; ^2^Cheeloo College of Medicine, Shandong University, Jinan, China; ^3^Department of Biostatistics, School of Public Health, Shandong University, Jinan, China; ^4^Katharine Hsu International Research Center of Human Infectious Diseases, Shandong Provincial Chest Hospital, Jinan, China; ^5^Department of Intensive Care Unit, Shandong Provincial Third Hospital, Jinan, China; ^6^College of Traditional Chinese Medicine, Shandong University of Traditional Chinese Medicine, Jinan, China

**Keywords:** tuberculosis, children, drug resistance, meta-analysis, national economic levels

## Abstract

**Background:** Drug-resistant tuberculosis (DR-TB), especially multidrug-resistant tuberculosis (MDR-TB) is a public health threat. Little is known about estimates of different profiles and rates of DR-TB among children globally.

**Methods:** We did a systematic review and meta-analysis of observational studies reporting DR-TB among children by searching Embase, PubMed, and Scopus databases from January 1, 2000 to October 1, 2020. Publications reporting more than 60 children with bacteriological confirmed tuberculosis and phenotypical drug susceptibility testing (DST) results were included. Pooled proportions of MDR-TB and sub-analysis by age subgroups, regions, economical levels were performed.

**Results:** We identified 4,063 studies, of which 37 were included. Of 23,652 pediatric TB patients, the proportions of DR-TB, MDR-TB, mono-resistant TB, polydrug resistant TB, extensively drug-resistant TB were 13.59% (1,964/14,453), 3.72% (881/23,652), 6.07% (529/8,719), 1.61% (119/7,361), 0.44% (30/6,763), respectively. The pooled proportion of MDR-TB among 23,652 children of 37 studies was 3.7% (95% CI, 3.5–4.0%). Rate of MDR-TB was much lower in high-income countries (1.8%) than that in lower-middle-income countries (6.3%) and upper-middle-income countries (7.3%). More specifically, the rates of MDR-TB were 1.7% in USA, 1.7% in UK, 2.9% in India, 6.0% in South Africa, 9.8% in China, respectively.

**Conclusions:** The burden of DR-TB remains high in children, and there are potential associations between rates of pediatric MDR-TB and national economical levels. More interventions on child TB cases in low-income countries may be urgently needed in future.

## Introduction

It is estimated that nearly 1 million children develop tuberculosis (TB) each year (1). However, most of these children are never diagnosed or treated for their disease, and there were an estimated 208,000 child deaths due to TB globally in 2019 (2). According to a mathematical modeling study, an estimation of 239,000 (95% confidence interval, CI: 194,000–298,000) children younger than 15 years died from tuberculosis worldwide in 2015, and 80% of these deaths were in children younger than 5 years (3). More than 70% of deaths occurred in the WHO southeast Asia and Africa regions (2, 3). Drug-resistant TB (DR-TB, referring to any resistance to TB drugs), especially multidrug-resistant TB (MDR-TB, defined as *Mycobacterium tuberculosis* with resistance to at least isoniazid and rifampicin), is a continuing threat in both children and adults, and more than 30,000 children had MDR-TB (2–4).

To our knowledge, few previous studies have focused on drug resistance patterns including mono-resistant tuberculosis (MR-TB), MDR-TB, polydrug resistant tuberculosis (PDR-TB), and extensively drug-resistant tuberculosis (XDR-TB) in children throughout the world, and very little is known about the magnitude of this disease and its drug resistance in children (5–7). The main reasons are as follows: Bacteriological confirmation of DR-TB diseases is more difficult to attain in children, because they are more likely to have paucibacillary and extrapulmonary disease than adults, and cannot expectorate sufficient sputum for laboratory evaluation (6–8). Moreover, children tend to swallow their sputum rather than expectorate it, and it was reported that sputum specimen of children are also more likely to become contaminated during collection (6). Therefore, diagnosis of TB in children is challenging despite the availability of modern technologies such as nucleic acid amplification tests. Clinical diagnostic criteria of pediatric TB is also not standardized (8, 9), microbiological confirmation of pulmonary TB succeeds in no more than 40% of children and even less frequently for extrapulmonary TB (7, 8). In brief, drug susceptibility testing results are often lacking in children, because current drug susceptibility testing (DST) was often done by genotypic methods either directly on specimens (e.g., Xpert MTB/RIF) or on cultured isolates (e.g., line probe assays) (7, 10).

Lack of detailed information on the distribution of pediatric MDR-TB globally would make it difficult for The WHO End TB Strategy to put forward specific TB control measures in local conditions (11). The regional distribution of tuberculosis disease varies greatly, and it was reported that in 2019, 30 high burden countries covered nearly 87% new cases of the global total (2). Whether MDR-TB among children were with high regional difference as well and which country had the highest risk of developing MDR-TB remains to be discussed. Recent years, economic levels especially social health spending was found to be related with TB mortality and incidence (12–14). Researchers have tried to create socio-economic models to predict trends of TB in different countries, but none of these studies have used the country's economic level as a measure for the prevalence/incidence of DR-TB cases (13).

We aim to describe the regional and global epidemiological characteristics of various types of DR-TB, especially MDR-TB, in children by doing a systematic review and meta-analysis, and to assess the scale of this problem and provide guidance on its global control. In addition, our study assessed the association between national economical levels and rates of MDR-TB among children, and discuss the regional differences of this disease.

## Materials and Methods

### Patient and Public Involvement

It was not appropriate or possible to involve patients or the public in the design, or conduct, or reporting, or dissemination plans of our research.

### Search Strategy and Selection Criteria

Two reviewers (SWM and LYF) searched Embase, PubMed, and Scopus to identify peer-reviewed articles reporting DR-TB in children from observational studies throughout the world published up from January 1, 2000 to October 1, 2020. All types of studies were searched. This study followed the Preferred Reporting Items for Systematic reviews and Meta-Analysis (PRISMA) guidelines (15). We used a Boolean search strategy with keywords and MeSH or Emtree terms pertaining to resistance, tuberculosis, and children for each database. Search terms were identified from relevant research, systematic reviews, reports, experts in pediatric tuberculosis, and the 2020 WHO TB report (2). A detailed summary of all search terms is provided in the Appendix Table 1.

SWM and LYF screened titles, abstracts, and full texts for inclusion, and differences were resolved by consensus of both authors. Eligible studies should report original results, with proportions of pediatric drug-resistant tuberculosis (for example, DR-TB, MR-TB, PDR-TB, MDR-TB, isoniazid-resistance, rifampicin-resistance, ethambutol-resistance, streptomycin-resistance or pyrazinamide-resistance) among more than 60 children (at least ≤ 19 years) TB patients confirmed by bacteriology and phenotypical DST results, to avoid small series reporting unusual cases. Reports from outbreak, clinical trials, case-control studies or contact investigations, where resistance among the included subset of patients is expected to be biased and less likely to represent resistance in the study base of all children with tuberculosis disease, were not included in our study. Comments, editorials, reviews, letters, case reports, and duplicate studies were also excluded. Only studies published in English were included.

### Quality Assessment and Data Extraction

The Agency for Healthcare Research and Quality (AHRQ) checklist was used to assess the quality of all selected cross-sectional and observational studies. AHRQ included 11 quality questions, each question can be answered by “yes,” “no,” and “unclear.” Low quality studies: 0–3 points, medium quality studies: 4–7 points, high quality studies: 7–11 points. Medium and high quality studies were included in our meta analysis. Quality assessment was conducted independently by two reviewers. Any disagreements between the two reviewers were resolved by discussion. The detailed information were provided in Appendix Table 2.

Two reviewers (SWM and LYF) manually screened the bibliographies of included articles to identify additional eligible studies. The authors of these eligible publications were contacted to retrieve the full texts when articles could not be accessed. Title and abstract screening, full text screening, data extraction, and quality assessment were also done independently by SWM and LYF. Any discrepancies were discussed until a consensus was reached. EndNote X9 was used as reference manager and to share references. Piloted forms were used for data extraction and quality assessment. Summary data were extracted on study design, country, screening setting, recruitment, population, sample size, age group, sample demographics, period of study, and reported phenotypical DST results.

### Definitions

First-line anti-TB drugs consist of isoniazid, rifampin, ethambutol, pyrazinamide and streptomycin. Drug-resistant tuberculosis (DR-TB) refers to any resistance to anti-TB drugs. Mono-resistance (MR) refers to resistance to one first-line anti-TB drug only. Multidrug resistance (MDR) refers to resistance to at least both isoniazid and rifampicin. Polydrug resistance (PDR) refers to resistance to more than one first-line anti-TB drug, other than both isoniazid and rifampicin. Extensively drug-resistant (XDR)-TB refers to MDR-TB plus resistance to a fluorquinolone and a second-line injectable agent (amikacin, kanamycin or capreomycin) (16). According to the World Bank, high-income economies are those that had a Gross national income (GNI) per capita of $12,535 or more in 2019 (17). Middle-income countries are those with $1,026–12,475 in per capita GNI (17). Upper middle-income countries refer to those with a GNI per capita between $4,046 and 12,535 (17). Lower middle-income countries refer to those with a GNI per capita between $1,036 and 4,045 (17). Countries enrolled in our meta-analysis including UK, USA, Canada, Spain, Switzerland, Norway, Korea, Germany were high-income economies, China, Thailand, Turkey, South Africa, Argentina were upper middle-income countries, and India, Egypt, Pakistan were lower middle-income countries (17).

### Data Analysis

To adjust for heterogeneity across the studies (e.g., across settings or different age-group populations), we used random-effects models (Der Simonian and Laird) for the analysis. Pooled estimates of the prevalence of MDR-TB were calculated for all pediatric TB cases across the included articles. We also did subgroup analysis (age subgroups, continent subgroup including North America/Europe/Asia/Africa, country subgroup including USA/India/China/South Africa/UK, national economic levels subgroup including lower-middle-income/upper-middle-income/high-income countries) for the pooled proportions of MDR-TB among pediatric patients.

In order to find specific study-level variables associated with the statistical heterogeneity between these studies, meta-regressions for variables including continents, country, national economical levels, publication year, age group were conducted (Appendix Table 3). In addition, heterogeneity was assessed through the use of the *I*^2^ statistic, *I*^2^ = 25% low heterogeneity, *I*^2^ = 50% moderate heterogeneity, *I*^2^ = 75% high heterogeneity (18). Heterogeneity was explored graphically in forest plots to determine whether characteristics such as age, study country, economic levels of their country across the studies might explain sources of heterogeneity, and we also investigated whether the subgroup analysis stratified by these factors would reduce heterogeneity.

Data were analyzed using Stata Version 15 (StataCorp, Texas). Metan, a Stata command, was used to calculate pooled estimates for proportion and 95% CIs. Metareg, a Stata command, was used to conduct meta-regressions.

## Results

### Search Results and Report Selection

**Figure 1:**
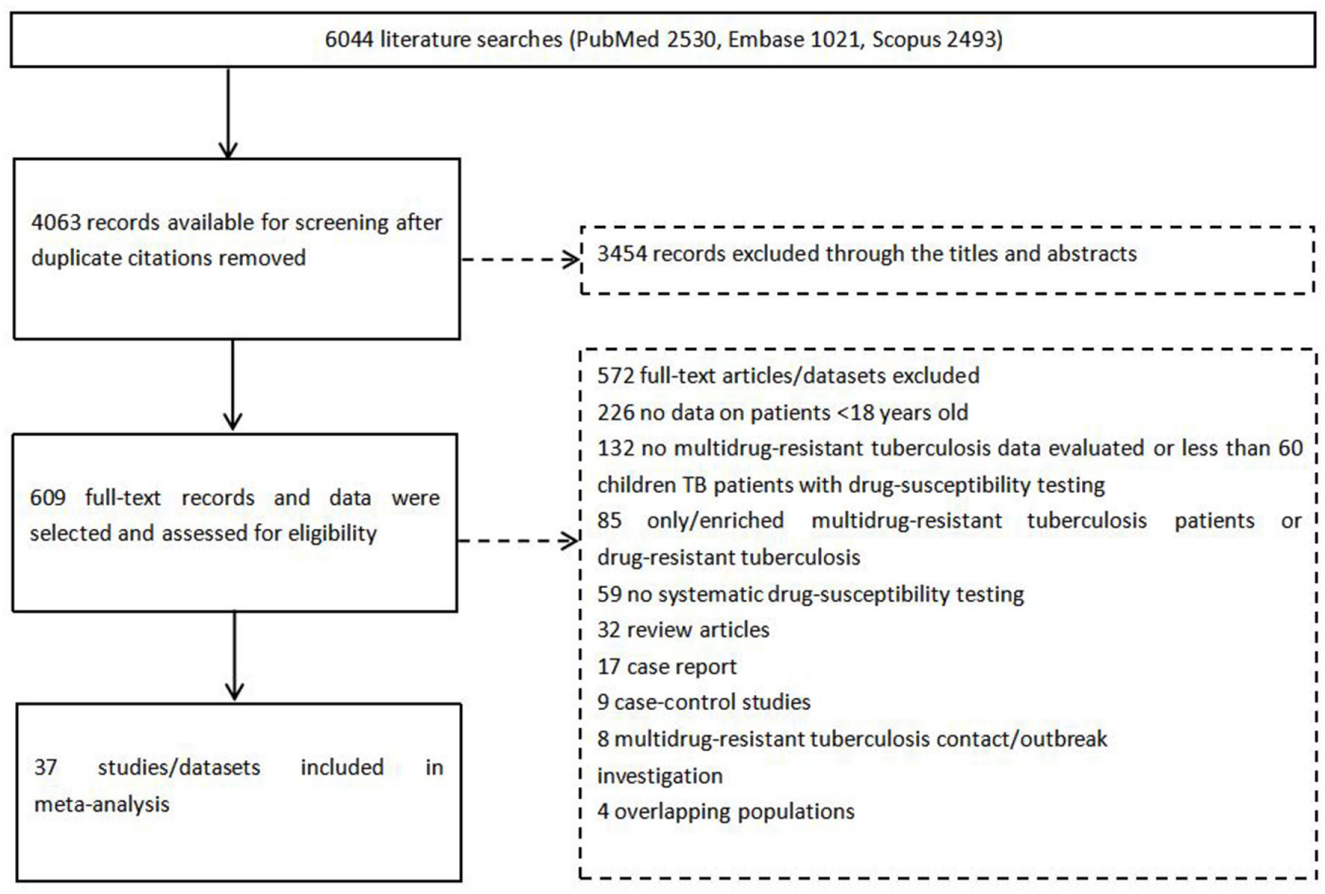
Flow chart for study selection. TB, tuberculosis.

The search yielded 4,063 reports; 609 records were selected through the titles after screening of abstracts and review of the full texts, 37 studies were eligible for inclusion (Figure 1).

### Report Characteristics

**Figure 2:**
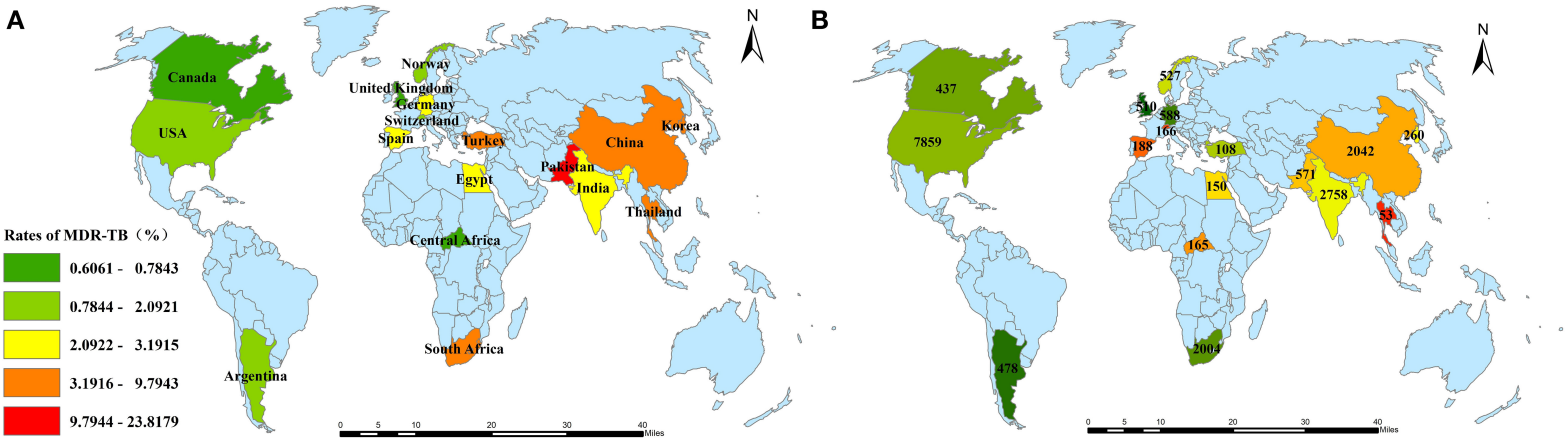
Geographic distribution. **(A)** the rates of MDR-TB in each country; **(B)** the number of pediatric TB cases with drug susceptibility results from each country. TB, tuberculosis; MDR-TB, multidrug resistant tuberculosis.

From January 1, 2000 to October 1, 2020, all eligible publications with a broad geographic distribution were collected to extract data sets (Figure 2), with the majority from USA, South Africa, India, China, USA, and UK. Table 1 illustrates the basic characteristics of our data. A total of 23,652 pediatric TB patients in these countries were included in the analysis. The proportions of DR-TB, MDR-TB, MR-TB, PDR-TB, XDR-TB among these patients were 13.59% (1,964/14,453), 3.72% (881/23,652), 6.07% (529/8,719), 1.61% (119/7,361), 0.44% (30/6,763), respectively. In addition, the drug-resistant rates of first-line anti-TB drugs, including isoniazid, rifampicin, ethambutol, streptomycin, pyrazinamide were 10.01% (1,104/11,032), 7.53% (559/7,421), 5.76% (313/5,431), 10.82% (497/4,594), 3.26% (120/3,685), respectively. Due to the heterogeneity of these studies included, the total population had to be divided into seven age groups including 0–12 years (total 175; DR-TB 22/175, 12.57%; MDR-TB 7/175, 4.00%), 0–13 years (total 1,856; DR-TB 198/1,532, 12.92%; MDR-TB 108/1,856, 5.82%), 0–14 years (total 7,207; DR-TB 669/5,359, 12.48%; MDR-TB 310/7,207, 4.30%), 0–15 years (total 8,888; DR-TB 627/3,813, 16.44%; MDR-TB 226/8,888, 2.54%), 0–16 years (total 3,293, DR-TB 196/2,456, 7.98%; MDR-TB 86/3,293, 2.61%), 0–18 years (total 1,706; DR-TB 356/1,706, 20.86%; MDR-TB 135/1,706, 7.91%), 0–19 years (total 527, MDR-TB: 9/527, 1.70%).

**Table 1:** Overview of 37 studies and 23,652 children tuberculosis cases with drug susceptibility results.

**References**	**Period**	**Location**	**Age (years)**	**TB (total)**	**Pan-susceptible TB**	**DR-TB**	**MDR-TB**	**MDR-TB (%)**	**MR-TB**	**PDR-TB**	**XDR-TB**	**Isoniazid-resistance**	**Rifampicin-resistance**	**Ethambutol-resistance**	**Streptomycin-resistance**	**Pyrazinamide-resistance**
Hasan et al. (19)	1990–2007	Pakistan	0–14	571			136	23.82								
Kassa-Kelembho et al. (20)	1998–2000	Central African Republic	0–15	165	140	25	1	0.61	18			15	0			
Jiao et al. (21)	2005–2012	China	0–18	259	129	130	57	22.01			4	77	67	56	90	
Seddon et al. (22)	2007–2009	South Africa	0–13	294	249	45	26	8.84				41	30			
Fairlie et al. (23)	2008	South Africa	0–14	148	125	23	13	8.78	9	1		21	15			
Cakir et al. (24)	2006–2010	Turkey	0–14	108	91	17	9	8.33				16	10	8	12	
Schaaf et al. (25)	2009–2011	South Africa	0–13	340	291	49	24	7.06				43	30			
Tao et al. (26)	2006–2015	China	0–18	784	636	148	54	6.89	67	27		95	65	43	112	
Schaaf et al. (27)	2005–2007	South Africa	0–13	285	242	43	19	6.67				41	29			
Wang et al. (28)	2010–2016	China	0–15	132	99	33	8	6.06	21	4		14	8	3	19	8
Kim et al. (29)	2007–2013	Korea	0–15	260	202	58	15	5.77			3	33	16	9	10	25
Lapphra et al. (30)	2008–2011	Thailand	0–18	53	38	18	3	5.66	13	2						
Schaaf (31)	2003–2005	South Africa	0–13	307	267	40	17	5.54				40	17			
Schaaf et al. (32)	2011–2013	South Africa	0–13	324			15	4.63				35	21			
Guo et al. (33)	2008–2013	China.	0–15	196	140	56	9	4.59				24	10	19	37	
Swaminathan et al. (34)	1995– 1997	India	0–12	175	153	22	7	4.00				22				
Prajapati et al. (35)	2009–2012	India	0–14	127	101	26	5	3.94	11			11	7			
Zhu et al. (36)	2000–2013	China	0–18	422	386	36	15	3.55								
Santiago et al. (37)	2005–2010	Spain	0–18	188	164	24	6	3.19				18				
van der Werf et al. (38)	2012	The European Union	0–14	410	356	54	13	3.17	34	7	0					
Shah and Shah (39)	2007–2013	India	0–16	1,311	1,225	86	39	2.97	6	7	6	84	73	52	69	40
Kodmon et al. (5)	2007–2015	The European Union	0–14	3,378	2,967	411	98	2.90	249	64	8					
Morcos et al. (40)		Egypt	0–15	150	113	37	4	2.67				8	4	2	10	
Shah and Shah (41)	2013–2016	India	0–16	1,145	1,035	110	28	2.45	8	7	9	80	70	59	54	42
Glasauer et al. (42)	2008–2017	Germany	0–14	588	484	104	14	2.38	65							
Schaaf et al. (43)	1994–1998	South Africa	0–13	306	285	21	7	2.29				21				
Abubakar et al. (44)	1999–2006	UK	0–16	837			19	2.27				78	20	7		5
Berberian et al. (45)	1998–2015	Argentina	0–15	478	432	46	10	2.09	28							
Oesch Nemeth et al. (46)	1996–2011	Switzerland	0–14	166			3	1.81								
Jensenius et al. (47)	1995–2014	Norway	0–19	527			9	1.71								
Smith et al. (48)	1993–2014	USA	0–15	4,826			82	1.70								
Granich et al. (49)	1994–2003	USA	0–14	601			10	1.66								
Nelson et al. (50)	1993–2001	USA	0–15	2,432	2,060	372	40	1.64				178				
Espinal et al. (51)		Others^*^	0–14	163	150	13	2	1.23								
Djuretic et al. (52)	1993–1999	UK	0–14	510			4	0.78				32				
Minion et al. (53)	1997–2008	Canada	0–14	437	416	21	3	0.69								
Jiao et al. (54)		China	0–15	249			57	22.89				77	67	55	84	

*TB, tuberculosis; DR-TB, drug-resistant tuberculosis; MR-TB, mono-resistant tuberculosis; MDR-TB, multidrug resistant tuberculosis; XDR-TB, extensively drug-resistant tuberculosis*.

* *Others refer to 11 countries including Bolivia, Dominican Republic, the Republic of Korea, Lesotho, Nepal, Peru, Portugal, Sierra Leone, Swaziland, Barcelona in Spain, and Shandong Province in the People's Republic of China*.

### Results of Meta-Regressions

As shown in Appendix Table 3, in the multivariate meta-regressions regarding the set of selected variables, continents (β = −0.017; S.E. = 0.010; *P* = 0.093) met the criterion of *P* < 0.10, but national economical levels (β = 0.008; S.E. = 0.008; *P* = 0.322), country (β = −0.001; S.E. = 0.002; *P* = 0.625), publication year (β = −0.002; S.E. = 0.002; *P* = 0.202), age group (β = 0.005; S.E. = 0.006; *P* = 0.457) did not met the criterion *P* < 0.10.

### Pooled Proportions of MDR-TB Among the Total Patients

**Figure 3:**
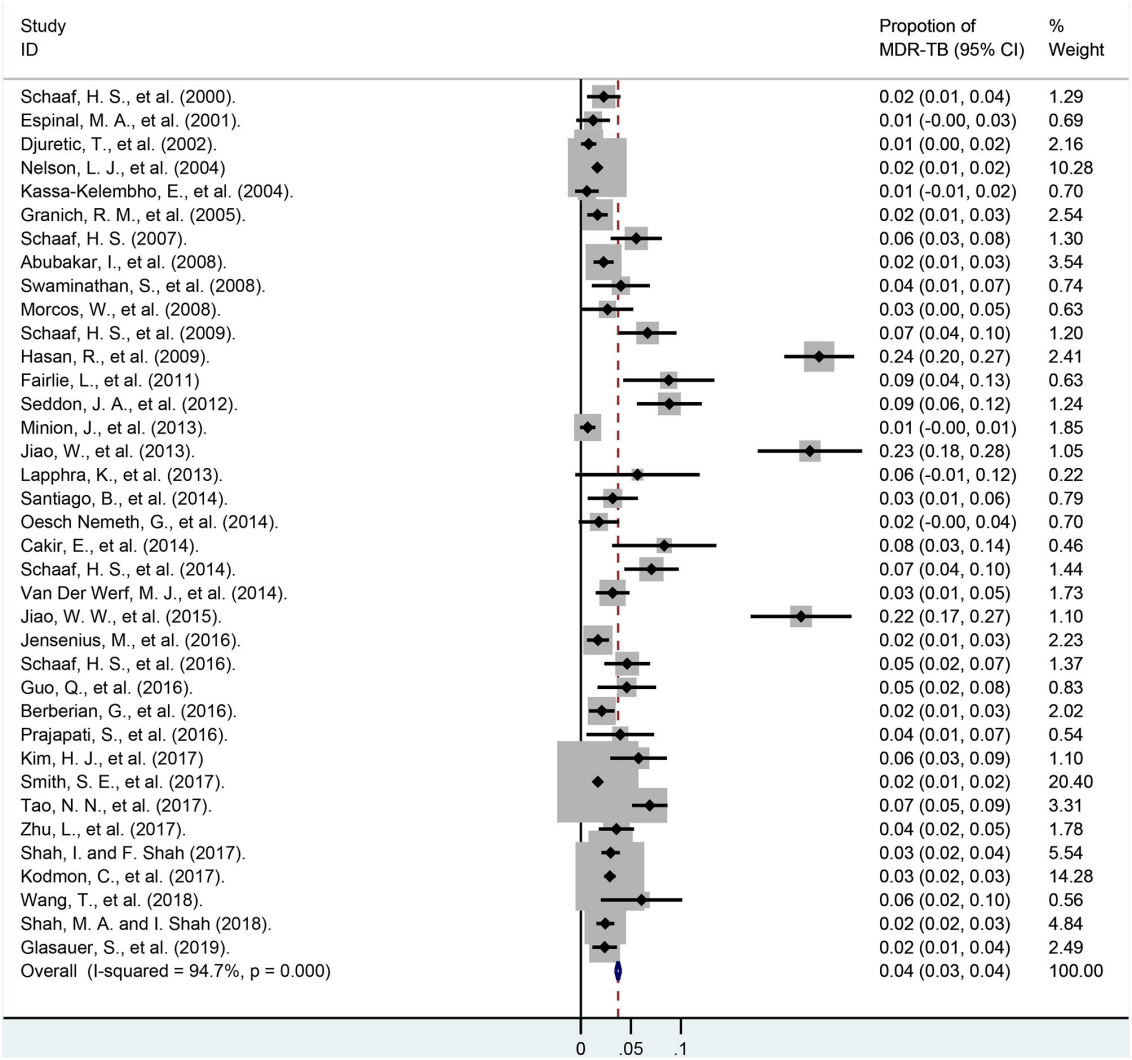
Forest plot depicting the pooled proportion of multidrug-resistant tuberculosis in children. MDR-TB, multidrug resistant tuberculosis; Horizontal lines represent the 95% CIs around the point estimates for each study and the gray shaded areas are proportional to the weight given to each study.

Rates of MDR-TB among total children are summarized in Figure 3 and Appendix Table 4. In our meta-analysis, the pooled proportion of MDR-TB among 23,652 children of 37 studies was 3.7% (95% CI, 3.5–4.0%). The ratio of MDR-TB varied from 0.6% (95% CI, −0.6–1.8%) to 23.8% (95% CI, 20.3–27.3%) between studies. The rates of MDR-TB among 24 studies were lower than 5.0%, and it was between 5.0 and 10.0% among another 10 studies. The last three studies were higher than 20%, and the weights of these three surveys among total cases were only 4.56%. Heterogeneity among the studies was high [*I*^2^ = 94.7%, degree of freedom (df) = 36, *P* < 0.001].

### Rates of MDR-TB Among Different Subgroups

**Figure 4:**
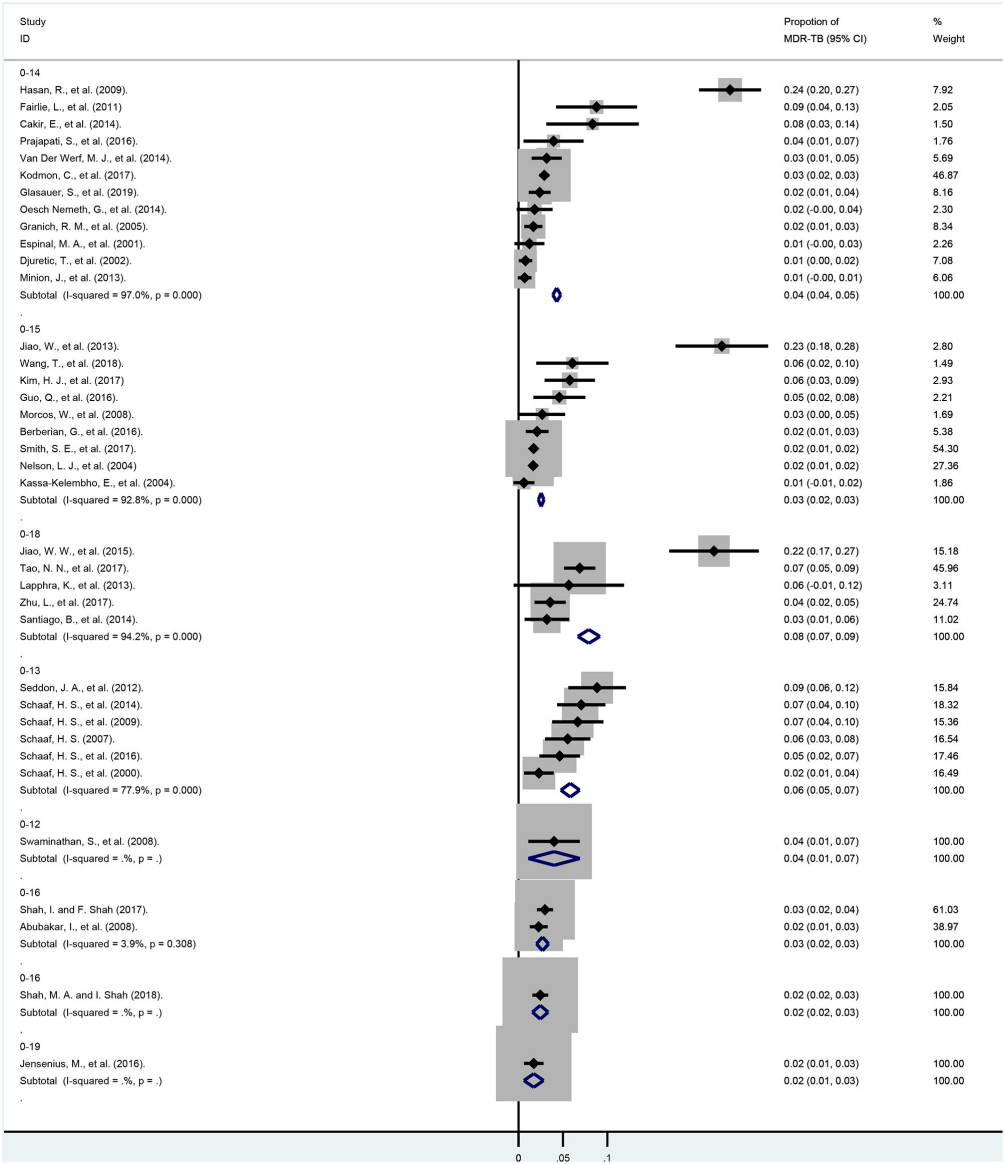
Forest plots for multidrug-resistant tuberculosis ratios from 37 studies in different age subgroups. MDR-TB, multidrug resistant tuberculosis; Horizontal lines represent the 95% CIs around the point estimates for each study and the gray shaded areas are proportional to the weight given to each study.

Subgroup analyses were based on age groups, regional distribution and the overall economic levels of each country. As shown in Figure 4 and Appendix Table 5, we found that when divided into different age groups, the pooled proportions of MDR-TB from smallest to largest were 1.7% (95% CI, 0.6–2.8%; df = 0; 0–19 years), 2.5% (95% CI, 2.2–2.9%; *I*^2^ = 92.8%, df = 8, *P* < 0.001, 0–15 years), 2.6% (95% CI, 2.1–3.2%; *I*^2^ = 0.0%, df = 2, *P* = 0.557, 0–16 years), 4.3% (95% CI, 3.9–4.7%; *I*^2^ = 97.0%, df = 11, *P* < 0.001, 0–14 years), 5.8% (95% CI, 4.8–6.9%; *I*^2^ = 77.9%, df = 5, *P* < 0.001, 0–13 years),7.9% (95% CI, 6.7–9.2%; *I*^2^ = 94.2%, df = 4, *P* < 0.001, 0–18 years), respectively.

**Figure 5:**
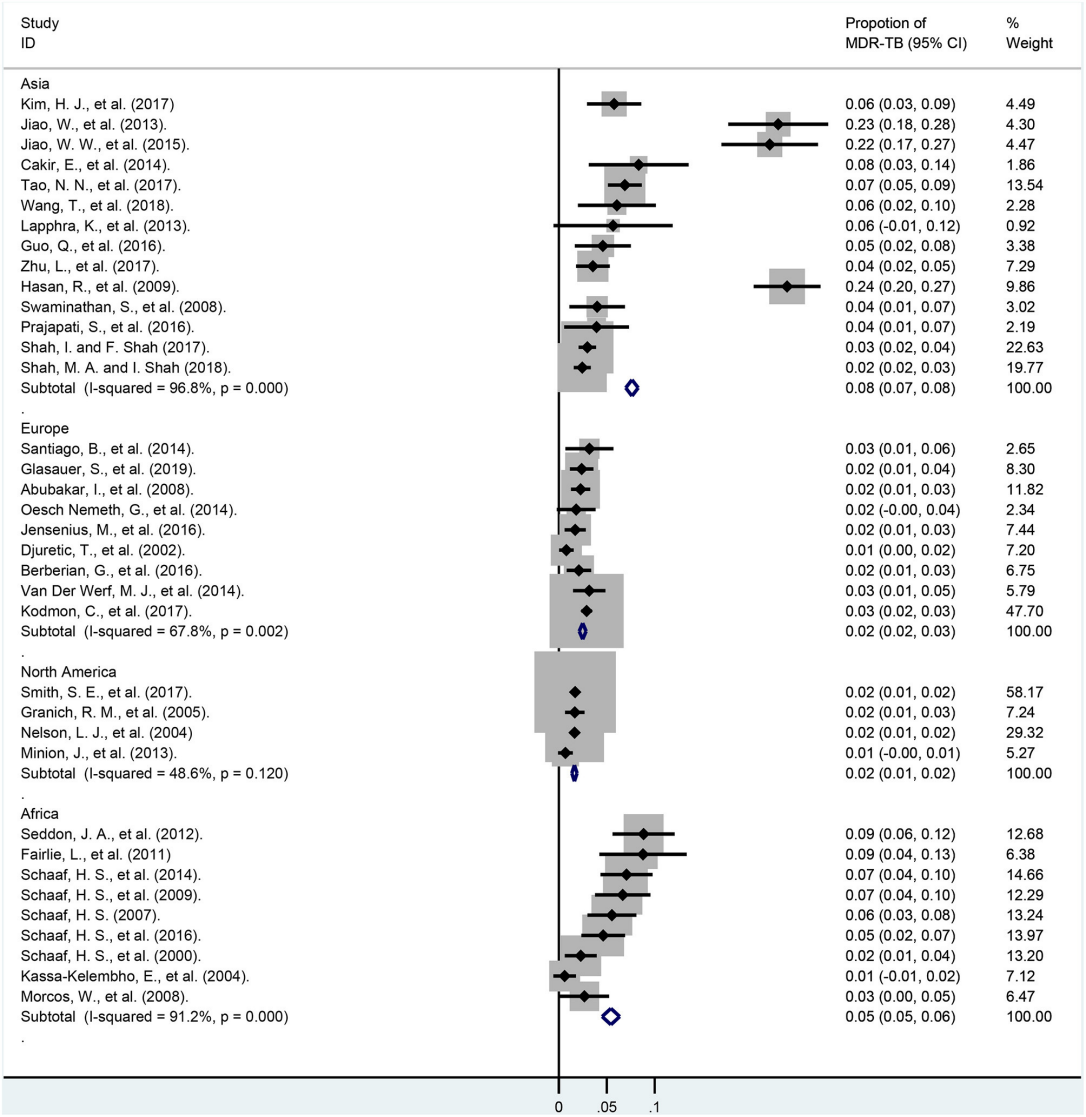
Forest plots for multidrug-resistant tuberculosis ratios in different continents. MDR-TB, multidrug resistant tuberculosis; Horizontal lines represent the 95% CIs around the point estimates for each study and the gray shaded areas are proportional to the weight given to each study.

In subgroup analyses of different continents (Figure 5; Appendix Table 6), the weights of the total cases from North America, Europe, Asia, and Africa were 35.08, 29.94, 24.49, 9.80%, respectively. Moreover, the rates of MDR-TB among these continents were 1.5% (95% CI, 1.1–1.9%; *I*^2^ = 46.4%, df = 3, *P* = 0.133; North America), 2.2% (95% CI, 1.5–2.8%; *I*^2^ = 63.5%, df = 8, *P* = 0.005; Europe), 4.9% (95% CI, 2.9–7.0%; *I*^2^ = 85.5%, df = 8, *P* < 0.001; Africa), 8.4% (95% CI, 5.8–11.0%; *I*^2^ = 94.9%, df = 13, *P* < 0.001; Asia), respectively.

**Figure 6:**
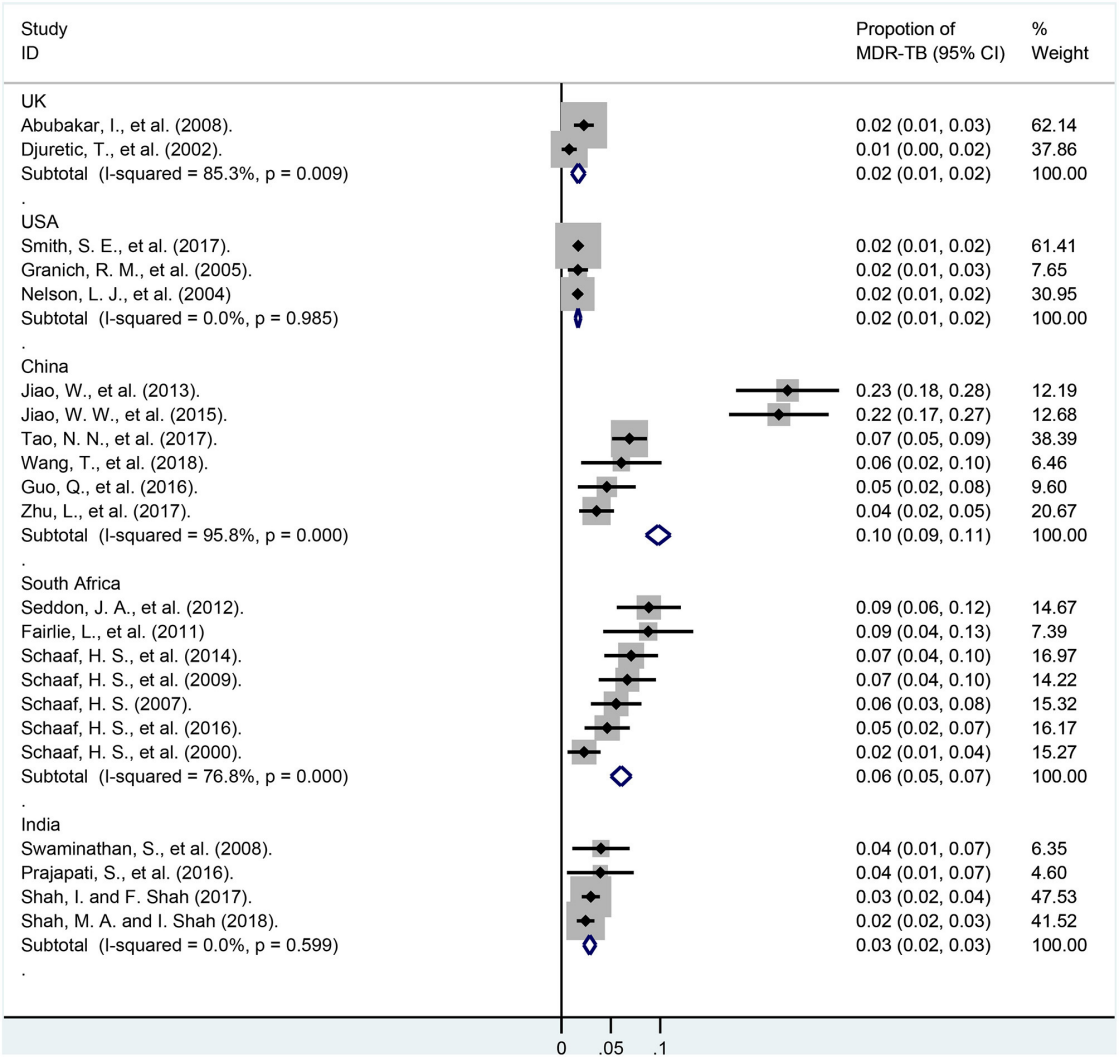
Forest plots for multidrug-resistant tuberculosis ratios in different country. MDR-TB, multidrug resistant tuberculosis; Horizontal lines represent the 95% CIs around the point estimates for each study and the gray shaded areas are proportional to the weight given to each study.

In subgroup analyses of various countries (Figure 6 and Appendix Table 7), the weights of the total cases from USA, India, China, South Africa, and UK were 33.23, 11.66, 8.63, 8.47, 5.70%, respectively. The proportions of MDR-TB among these countries were 1.7% (95% CI, 1.4–2.0%; *I*^2^ = 0.0%, df = 2, *P* = 0.985; USA), 1.7% (95% CI, 1.0–2.4%; *I*^2^ = 85.3%, df = 1, *P* = 0.009; UK), 2.9% (95% CI, 2.2–3.5%; *I*^2^ = 0.0%, df = 3, *P* = 0.599; India), 6.0% (95% CI, 5.0–7.1%; *I*^2^ = 95.8%, df = 5, *P* < 0.001; South Africa), 9.8% (95% CI, 8.5–11.0%; *I*^2^ = 95.80%, df = 5, *P* < 0.001; China), respectively.

**Figure 7:**
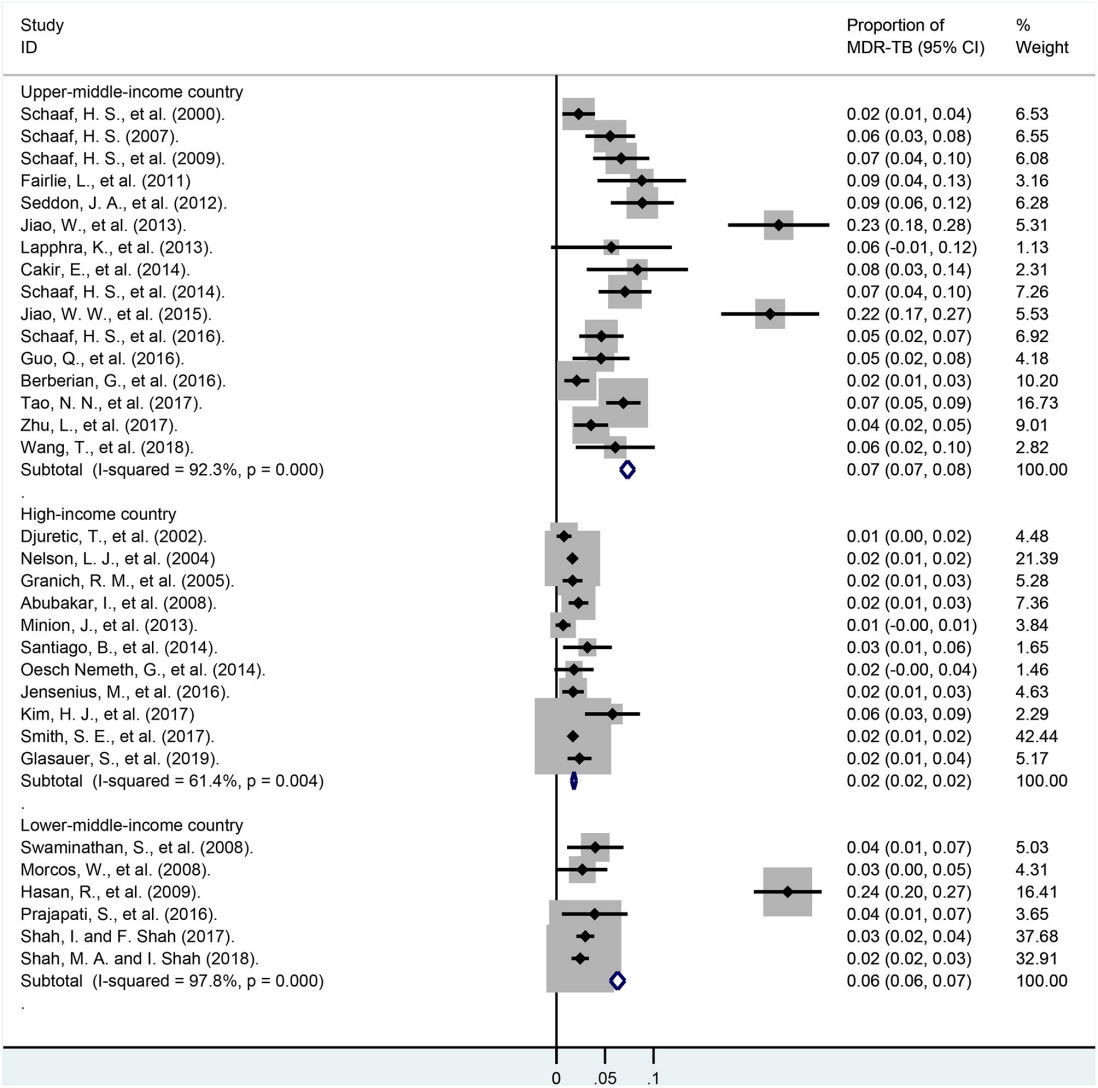
Forest plots for multidrug-resistant tuberculosis ratios in lower-middle-income, upper-middle-income, high-income countries. MDR-TB, multidrug resistant tuberculosis; Horizontal lines represent the 95% CIs around the point estimates for each study and the gray shaded areas are proportional to the weight given to each study.

These pediatric patients were divided into different subgroups according to the economic levels of their country (Figure 7; Appendix Table 8). In lower-middle-income and upper-middle-income countries, the pooled proportions of MDR-TB were 6.3% (95% CI, 5.5–7.1%; *I*^2^ = 97.8%, df = 5, *P* < 0.001; weight, 14.71%) and 7.3% (95% CI, 6.6–8.0%; *I*^2^ = 92.3%, df = 15, *P* < 0.001; weight, 19.81%). However, it was much lower in these high-income countries, and was 1.8% (95% CI, 1.6–2.0%; *I*^2^ = 61.4%, df = 10, *P* = 0.004; weight, 48.08%).

## Discussion

The findings from our meta-analysis highlight and quantify the burden of drug-resistance (including DR-TB, MDR-TB, MR-TB, PDR-TB, XDR-TB, and any first-line anti-TB drug resistance) among pediatric TB patients around the world by calculating the pooled proportions in different subgroups stratified by age, continents, countries and national economic levels. In general, pediatric TB cases from North America, and Europe had a much lower rate of MDR-TB than those from Africa and Asia. In addition, we found that high economic levels of country might be associated with less drug resistance, for instance, the pooled proportion of MDR-TB from high-income countries were less than one third of lower-middle-income and upper-middle-income countries. We also found that previous drug resistance surveillance studies in children with TB were mainly done in the age groups 0–14 and 0–15 years of age.

Globally, only a few studies (7, 55, 56) investigated the rates of pediatric DR-TB, and most were merely focused on MDR-TB or RR-TB but not including other drug-resistant subgroups. For example, a country-level, regional, and global estimates of DR-TB in children estimated that 850,000 children developed tuberculosis in 2014, among which ~6.82% were isoniazid-monoresistant tuberculosis, 2.94% were MDR-TB and 0.14% with XDR-TB (56). As a supplementary for the epidemiology on DR-TB among children, we found that the proportions of DR-TB, MDR-TB, MR-TB, PDR-TB, XDR-TB globally were 13.59, 3.72, 6.07, 1.61, 0.44%, respectively. The estimated burden of DR-TB cases indicates a vast gap between the resistance rates and treatment (5, 7, 56). According to the WHO Global TB Report of 2020 (2), an estimated 3.4% (95% CI, 2.5–4.4%) of new cases and 18% (95% CI, 7.6–31%) of previously treated cases had MDR/RR-TB among overall TB cases from all age groups. However, there remains a lack of detailed information on epidemiology and drug resistance profiles among pediatric TB cases. Children accounted for 12% of all TB cases in 2019, compared with 32% of cases in adult women and 56% in adult men (2). Although children population usually represent a relatively small fraction of total TB patients, a particular vulnerability to severe disease and death following infection such as the acquisition of multidrug resistance makes it a great health burden to the world (2, 3). Thus, further studies into the epidemiology, immune mechanisms, diagnosis, treatment, and prevention of childhood DR-TB are urgently needed (3–7, 10).

Our study helps to figure out the areas at high risk of pediatric MDR-TB; the proportions of MDR-TB among child TB cases from high to low are distributed in Pakistan (23.8%), China (9.8%), South Africa (6.0%), India (2.9%), USA (1.7%), UK (1.7%). Interestingly, the distribution of pediatric MDR-TB was to some degree consistent with the epidemic of total TB cases. In 2019, eight high TB burden countries accounted for two thirds of the global total: six Asian countries including India (26%), Indonesia (8.5%), China (8.4%), the Philippines (6.0%), Pakistan (5.7%), Bangladesh (3.6%), and two African countries including Nigeria (4.4%), South Africa (3.6%) (2). The characteristics of its distribution may be contributed by the differences on socioeconomic determinants and health system development between these countries (12–14). During our study, we took the economic levels of each country into account to make a subgroup analysis, and found that the pooled proportions of MDR-TB among children was much higher in these middle-income countries (6.3 and 7.3%) than in high-income countries (1.8%). More interventions such as improving BCG vaccine coverage, conducting health education on TB prevention, cleaning and disinfecting school classrooms regularly enforcing the TB monitoring among children, directly observed treatment, short-course (DOTS) strategy to increase treatment compliance, increasing access to public health services may be urgently needed to control child TB cases in low-income countries in future.

Actually, there were already some studies majored in the impact of socioeconomic determinants, especially public health spending, on tuberculosis case detection, tuberculosis prevalence, all-cause tuberculosis mortality rate, and tuberculosis treatment success rates (12–14, 57). A cross-national statistical modeling analysis in 21 European countries, 1995–2012 found that each US$100 increase in social protection spending was associated with a decrease per 100,000 population in the number of tuberculosis case notifications of −1.53%, estimated incidence rates of −1.70%, non-HIV-related tuberculosis mortality rate of −2.74%, and all-cause tuberculosis mortality rate of −3.08% (14). In spite of the abundant research achievements on the association between economics and tuberculosis, none assessed whether a strict relation exists between socioeconomic determinants and drug resistance rate in TB patients (12–14, 57). Therefore, our findings may offer a theoretical foundation and practical experience of further investigations in the field of DR-TB and economical levels. It is possible that in near future, human development index, corruption perception index, Gini coefficient, gross domestic product (GDP) per capita and countries, and other economic indicators may be taken into consideration to evaluate and forecast the rates of MDR-TB among children population (13).

Although the number and incidence of TB cases were highest in the WHO South-East Asia and Africa regions, accounting for more than two thirds of the global burden (58), the number of child tuberculosis cases with phenotypical DST results in Asia and Africa regions that were extracted from the literature enrolled was much less than that in USA and Europe. The weights of effective data from Asia and Africa in our meta-analysis made up only 34.28% of the total. There are several explanations: First, the lower detection rate of MDR-TB in Africa and Asia may contribute. DST is not routinely available among TB patients, and only some high-income countries can afford to do DST of first-line anti-TB drugs in all primary TB patients (59, 60). Second, other factors including the differentiation on the diagnostic level of pediatric TB, publication bias, and the lack of high-risk screening program were also to blame (2, 57). There is still a long way to go to monitor and control tuberculosis as well as its drug resistance among children (2, 55).

This study has some strengths. First, we described the crude ratio of drug-resistant subgroups including DR-TB, MDR-TB, MR-TB, PDR-TB, XDR-TB, and any first-line anti-TB drug resistance, which was an improvement over previous studies (5, 7, 56), and could provide guidance on a global level for the burden of drug-resistant tuberculosis in all types among children. Second, to a certain extent, this was innovation on subgroup analysis by age groups, countries, continents and national economic levels. Third, this meta-analysis was based on the phenotypical DST results of 23,652 pediatric TB patients and was more representative. Finally, this framework of drug resistance profiles would provide a reference for clinicians when choosing the anti-TB drugs.

This study also shares several limitations with the companion studies (7, 61, 62). First, detailed demographic information on each TB cases were not described due to the heterogeneity of the publications enrolled, and the indicators in most of these studies were inconsistent. Second, the data in our study were extracted from previous publications, and were limited compared with from data from prospective census-based cohort study. Third, although subgroup analysis were already made, the pooled proportions of MDR-TB were still with high heterogeneity, similar to most previous meta-analysis majoring in rates including incidence, mortality, drug resistance rate and others (61, 62). Thus, the potential factors that affected the rates of MDR-TB among children remains to be figured out in future. Finally, although phenotypical DST results including at least isoniazid, rifampin, ethambutol, and streptomycin, it is possible that these child TB cases are potentially resistant to other drugs especially second-line anti-TB drugs they did not tested.

## Conclusion

This meta-analysis suggests that the burden of DR-TB in children is largely unknown and often unreported (2, 63), the estimations on the proportions of DR-TB (13.59%), MDR-TB (3.72%), MR-TB (6.07%), PDR-TB (1.61%), XDR-TB (0.44%), isoniazid-resistance (10.01%), rifampicin-resistance (7.53%), ethambutol-resistance (5.76%), streptomycin-resistance (10.82%), pyrazinamide-resistance (3.26%) among child cases globally could provide more guidance for WHO TB control. In addition, our findings also indicate the potential association between rates of pediatric MDR-TB and national economic levels, thus more interventions on child TB cases from low-income countries may be urgently needed. Actually, in order to improve the identification of MDR-TB and decrease its mortality among children, routine surveillance activities focused on this susceptible population should be systematically carried out in future including drug resistance surveys when routine surveillance is not in place.

## Data Availability Statement

The original contributions presented in the study are included in the article/Supplementary Material, further inquiries can be directed to the corresponding authors.

## Ethics Statement

The protocols applied in this study were approved by the Ethics Committee of Shandong Provincial Hospital, affiliated with Shandong University (SPH) and the Ethic Committee of Shandong Provincial Chest Hospital (SPCH), China. Consents from the participants were not required due to the anonymous nature of the data.

## Author Contributions

J-yL, W-mS, and Y-fL conceived and designed the study. H-cL and C-bY directed its implementation including the data analysis and writing of the paper. W-mS and Y-fL analyzed the data. YL, J-yL, Y-xL, and Y-fL contributed materials/analytic tools. W-mS and H-cL wrote and revised the manuscript. All authors revised it critically for important intellectual content, gave final approval of the version to be published and agreed to be accountable for all aspects of the work in ensuring that questions related to the accuracy or integrity of any part of the work were appropriately investigated and resolved.

## Conflict of Interest

The authors declare that the research was conducted in the absence of any commercial or financial relationships that could be construed as a potential conflict of interest.

## Publisher's Note

All claims expressed in this article are solely those of the authors and do not necessarily represent those of their affiliated organizations, or those of the publisher, the editors and the reviewers. Any product that may be evaluated in this article, or claim that may be made by its manufacturer, is not guaranteed or endorsed by the publisher.

## Abbreviations

TB: tuberculosis
DR-TB: drug-resistant tuberculosis
WHO: world health organization
RR-TB: rifampin-resistant tuberculosis
MR-TB: mono-resistant tuberculosis
MDR-TB: multi-resistant tuberculosis
PDR-TB: polydrug resistant tuberculosis
XDR-TB: extensive drug resistant TB
ORs: odds ratios
CI: confidence interval
MTB: *mycobacterium tuberculosis*
DST: drug susceptibility testing
DOTS, observed treatment: short-course strategy.
