# Supplementary material for: Drug-Resistant Tuberculosis Among Children: A Systematic Review and Meta-Analysis
Source: Front Public Health. 2021 Aug 18;9:721817. doi: 10.3389/fpubh.2021.721817 (PMC8416474; doi:10.3389/fpubh.2021.721817)
Supplement: Supplementary file 2 [file Table_2.DOC]

Appendix table 1 Search strategies for PubMed, Embase, and Scopus

|  | **Database: Pubmed; Search date: 01-October 2020** |  |
| --- | --- | --- |
| Search | Query | Items found |
| #1 | Search ( ((((tuberculosis, multidrug-resistant[mh] OR multidrug resistant tuberculosis[tiab] OR drug resistant tuberculosis[tiab] OR multiple drug resistant tuberculosis[tiab] OR MDR tuberculosis[tiab] OR MDR TB[tiab] OR MDRTB[tiab] OR ((drug resistance[tiab] OR multidrug resistance[tiab] OR multiple drug resistance[tiab] OR multiresistant[tiab] OR multi resistant[tiab]) AND (tuberculosis[tiab] OR TB[tiab]))))) AND (((infant[mh] OR infant[tiab] OR infants[tiab] OR infancy[tiab] OR toddler*[tiab] OR preterm*[tiab] OR prematur*[tiab] OR postmatur*[tiab] OR baby[tiab] OR babies[tiab] OR neonat*[tiab] OR newborn[tiab] OR preschool*[tiab] OR pre-school*[tiab] OR child[mh] OR child*[tiab] OR kindergar*[tiab] OR pupil*[tiab] OR schoolchild*[tiab] OR teen*[tiab] OR youth[tiab] OR youths[tiab] OR youngster*[tiab] OR young person*[tiab] OR young people[tiab] OR minors[mh] OR minors[tiab] OR puberty[mh] OR puberty[tiab] OR pubescen*[tiab] OR prepubescen*[tiab] OR paediatric*[tiab] OR pediatric*[tiab] OR peadiatric*[tiab] OR schools[mh:noexp] OR school*[tiab] OR kid[tiab] OR kids[tiab] OR boy*[tiab] OR girl*[tiab] OR creche*[tiab] OR highschool*[tiab] OR juvenil*[tiab] OR adolescent[mh] OR adolescen*[tiab] OR under ag*[tiab] OR underage*[tiab])))) AND (("2000/01/01"[Date - Publication] : "2020/10/01"[Date - Publication])) | 2530 |
|  | **Database: Embase; Search date: 01-October 2020** |  |
| No. | Query | Results |
| #1 | (infant:ti,ab,kw OR infants:ti,ab,kw OR infancy:ti,ab,kw OR toddler:ti,ab,kw OR preterm:ti,ab,kw OR prematur:ti,ab,kw OR postmatur:ti,ab,kw OR baby:ti,ab,kw OR babies:ti,ab,kw OR neonat:ti,ab,kw OR newborn:ti,ab,kw OR preschool:ti,ab,kw OR child:ti,ab,kw OR kindergar:ti,ab,kw OR pupil:ti,ab,kw OR schoolchild:ti,ab,kw OR teen:ti,ab,kw OR youth:ti,ab,kw OR youths:ti,ab,kw OR youngster:ti,ab,kw OR 'young person':ti,ab,kw OR 'young persons':ti,ab,kw OR 'young people':ti,ab,kw OR minors:ti,ab,kw OR puberty:ti,ab,kw OR pubescen:ti,ab,kw OR prepubescen:ti,ab,kw OR paediatric:ti,ab,kw OR pediatric:ti,ab,kw OR kid:ti,ab,kw OR school:ti,ab,kw OR boy:ti,ab,kw OR girl:ti,ab,kw OR creche:ti,ab,kw OR highschool:ti,ab,kw OR juvenile:ti,ab,kw OR juvenil:ti,ab,kw OR adolescent:ti,ab,kw OR adolescen:ti,ab,kw OR underage:ti,ab,kw OR children:ti,ab,kw OR childhood:ti,ab,kw) AND ((tuberculosis:ti,ab,kw OR tb:ti,ab,kw) AND ('drug resistance':ti,ab,kw OR 'multidrug resistance':ti,ab,kw OR 'multiple drug resistance':ti,ab,kw OR multiresistant:ti,ab,kw) OR 'drug resistant tuberculosis':ti,ab,kw OR 'multidrug resistant tuberculosis':ti,ab,kw OR 'extensively drug resistant tuberculosis':ti,ab,kw OR 'resistant tuberculosis':ti,ab,kw OR 'mdr tb':ti,ab,kw OR 'xdr tb':ti,ab,kw OR 'pdr tb':ti,ab,kw OR 'mr tb':ti,ab,kw OR 'dr tb':ti,ab,kw) AND [2000-2020]/py | 1021 |
|  | **Database: Scopus; Search date: 01-October 2020** |  |
| No. | Query | Results |
| #1 | ( TITLE-ABS-KEY ( tuberculosis )  AND  TITLE-ABS-KEY ( resistant )  AND  TITLE-ABS-KEY ( "child"  OR  "children"  OR  pediatric ) ) | 2493 |

**Appendix Table 2** Agency for Healthcare Research and Quality (AHRQ) checklist for assessing the quality of observational studies

| Study | 1 | 2 | 3 | 4 | 5 | 6 | 7 | 8 | 9 | 10 | 11 |
| --- | --- | --- | --- | --- | --- | --- | --- | --- | --- | --- | --- |
| Hasan, R., et al. (2009).[28] | + | + | + | + | U | - | + | - | - | + | U |
| Kassa-Kelembho, E., et al. (2004).[29] | + | + | + | + | U | + | + | - | U | - | U |
| Jiao, W. W., et al. (2015).[30] | + | + | + | + | U | - | + | - | - | - | U |
| Seddon, J. A., et al. (2012).[31] | + | + | + | + | U | U | + | U | - | + | U |
| Fairlie, L., et al. (2011).[32] | + | + | + | + | U | + | U | - | + | + | U |
| Cakir, E., et al. (2014).[33] | + | + | + | + | U | - | + | U | - | U | U |
| Schaaf, H. S., et al. (2014).[34] | + | + | + | + | U | - | - | + | U | U | + |
| Tao, N. N., et al. (2017).[35] | + | + | + | + | U | + | + | - | U | + | - |
| Schaaf, H. S., et al. (2009).[36] | + | + | + | + | U | - | - | + | U | U | + |
| Wang, T., et al. (2018).[37] | + | + | + | + | U | + | + | - | U | + | - |
| Kim, H. J., et al. (2017).[38] | + | + | + | + | U | - | + | - | + | U | U |
| Lapphra, K., et al. (2013).[39] | + | + | + | + | U | + | + | U | + | - | U |
| Schaaf, H. S. (2007).[40] | + | + | + | + | U | - | - | + | U | U | + |
| Schaaf, H. S., et al. (2016).[41] | + | + | + | + | U | - | - | + | U | U | + |
| Guo, Q., et al. (2016).[42] | + | + | + | + | U | + | + | - | U | + | - |
| Swaminathan, S., et al. (2008).[43] | + | + | + | + | U | - | + | - | + | - | + |
| Prajapati, S., et al. (2016).[44] | + | + | + | + | U | + | + | - | U | - | U |
| Zhu, L., et al. (2017).[45] | + | + | + | + | U | + | + | - | U | + | - |
| Santiago, B., et al. (2014).[46] | + | + | + | + | U | - | + | - | + | U | U |
| Van Der Werf, M. J., et al. (2014).[47] | + | + | + | + | U | + | U | U | U | + | U |
| Shah, I. and F. Shah (2017).[48] | + | + | + | + | U | - | + | - | + | U | U |
| Kodmon, C., et al. (2017).[5] | + | + | + | + | U | + | U | U | U | + | U |
| Morcos, W., et al. (2008).[49] | + | + | - | + | U | - | + | - | + | - | U |
| Shah, M. A. and I. Shah (2018).[50] | + | + | + | + | U | - | + | - | U | U | U |
| Glasauer, S., et al. (2019).[51] | + | + | + | + | U | - | + | - | + | - | U |
| Schaaf, H. S., et al. (2000).[52] | + | + | + | + | U | - | - | + | U | U | + |
| Abubakar, I., et al. (2008).[53] | + | + | + | + | U | - | U | - | + | - | U |
| Berberian, G., et al. (2016).[54] | + | + | + | + | U | U | U | U | + | - | U |
| Oesch Nemeth, G., et al. (2014).[55] | + | + | + | + | U | - | U | - | + | - | U |
| Jensenius, M., et al. (2016).[56] | + | + | + | + | U | U | U | - | + | - | U |
| Smith, S. E., et al. (2017).[57] | + | + | + | + | U | + | + | + | + | - | U |
| Granich, R. M., et al. (2005).[58] | + | + | + | + | U | U | U | U | + | - | U |
| Nelson, L. J., et al. (2004).[59] | + | + | + | + | U | - | U | - | + | - | U |
| Espinal, M. A., et al. (2001).[60] | + | + | - | + | U | + | U | - | U | - | U |
| Djuretic, T., et al. (2002).[61] | + | + | + | + | U | + | + | - | U | - | U |
| Minion, J., et al. (2013).[62] | + | + | - | + | U | - | + | - | + | - | U |
| Jiao, W., et al. (2013).[63] | + | + | + | + | U | - | + | - | + | - | U |
| **Notes:** Yes= +, No= -； U= unclear  1)Define the source of information (survey,record review). 2)List inchusion and exchusion criteria for exposed and umexposed subjects (cases and controls) or refer to previous publications. 3)Indicate time period used for identifying patients. 4)Indicate whether or not subjects were consecutive if not population-based. 5)Indicate if evaluators of subjective components of study were masked to other aspects of the status of the participants. 6)Describe any assessments undertaken for quality assurance purposes (e.g.,testretest of primary outcome measurements) 7)Explain any patient exchusions from analysis. 8)Describe how confoumding was assessed and'or controlled. 9)If applicable,explain how missing data were handled in the analysis. 10)Summarize patient response rates and completeness of data collection. 11)Clarify what follow-up,ifany,was expected and the percentage ofnatients for which incomnlete data or follow-up. | | | | | | | | | | | |
|
|
|
|
|
|
|
|
|
|
|
|
|

**Appendix Table 3** Results of meta-regression
