# Supplementary material for: Drug-Resistant Tuberculosis Among Children: A Systematic Review and Meta-Analysis
Source: Front Public Health. 2021 Aug 18;9:721817. doi: 10.3389/fpubh.2021.721817 (PMC8416474; doi:10.3389/fpubh.2021.721817)
Supplement: Supplementary file 3 [file Table_3.DOC]

**Appendix Table 4** The pooled proportion of multidrug-resistant tuberculosis in children

**Appendix Table 5** The pooled ratios of multidrug-resistant tuberculosis from 37 studies in different age subgroups

**Appendix Table 6** The pooled ratios of multidrug-resistant tuberculosis in different [continent](../../../../C:/Users/17865/AppData/Local/youdao/dict/Application/8.9.5.0/resultui/html/index.html#/javascript:;)s

**Appendix Table 7** The pooled ratios of multidrug-resistant tuberculosis in different country

**Appendix Table 8** The pooled ratios of multidrug-resistant tuberculosis in lower-middle-income, upper-middle-income, high-income countries
